# Secondhand Smoke Exposure and Cognitive Function in U.S. Never‐Smoking Older Adults

**DOI:** 10.1002/alz70860_099169

**Published:** 2025-12-23

**Authors:** Sophie Zhijing Xu, Jinyu Hu, Jackie Finik, Yaguang Zheng, Bei Wu

**Affiliations:** ^1^ New York University, New York, NY, USA; ^2^ NYU Aging Incubator, New York, NY, USA

## Abstract

**Background:**

While active smoking is a recognized risk factor for cognitive decline, the impact of secondhand smoke (SHS) exposure on cognitive function in never‐smoking adults remains understudied and inconsistent. This study investigates the association between SHS exposure and cognitive function in never‐smoking older adults in the U.S.

**Method:**

We analyzed data from 1,369 adults aged 60 years and older from the National Health and Nutrition Examination Survey 2011–2014. SHS exposure was quantified using serum cotinine levels and categorized as low exposure (0.01–0.02 ng/mL), moderate exposure (0.02–0.04 ng/mL), and high exposure (≥0.04 ng/mL). Participants who reported smoking fewer than 100 cigarettes in their lifetime were classified as never‐smokers. Cognitive function was assessed using the CERAD‐WL immediate and delayed recall tests, the Animal Fluency Test (AFT), and the Digit Symbol Substitution Test (DSST). Multivariable regression models were used to examine the association between SHS exposure and cognitive function, adjusting for demographic, lifestyle, and socioeconomic covariates.

**Result:**

The mean age was highest in the low SHS exposure group (70.37 ± 6.97 years), followed by the moderate (68.78 ± 6.69 years) and high (68.35 ± 6.60 years) exposure groups. The low exposure group had the highest proportion of individuals with some college education (61%), compared to 47% in the moderate group and 45% in the high exposure group. The high exposure group had the largest proportion of individuals with a low income‐to‐poverty ratio (27%). Compared to the low exposure group, participants in the moderate exposure group showed significantly lower scores in CERAD‐WL immediate recall (β = ‐1.69, *p* < 0.01), CERAD‐WL delayed recall (β = ‐0.88, *p* < 0.05), AFT (β = ‐1.76, *p* < 0.01), and DSST (β = ‐3.55, *p* < 0.05). No significant associations were observed in the high SHS exposure group across all cognitive tests.

**Conclusion:**

Moderate SHS exposure was significantly associated with worse cognitive performance in never‐smoking older adults, highlighting the cognitive risks of even limited SHS exposure. No significant associations were observed in the high exposure group, possibly due to sample size limitations or unmeasured confounders. Public health efforts to reduce SHS exposure are critical for preserving cognitive health in older adults.

